# Solution-Processed Hybrid Europium (II) Iodide Scintillator for Sensitive X-Ray Detection

**DOI:** 10.34133/research.0125

**Published:** 2023-05-05

**Authors:** Xue Zhao, Pengfei Fu, Pan Li, Hainan Du, Jinsong Zhu, Ciyu Ge, Longbo Yang, Boxiang Song, Haodi Wu, Tong Jin, Qingxun Guo, Liang Wang, Jinghui Li, Zewen Xiao, Jingjing Chang, Guangda Niu, Jiajun Luo, Jiang Tang

**Affiliations:** ^1^ School of Microelectronics, Xidian University, Xi’an 710071, China.; ^2^ Wuhan National Laboratory for Optoelectronics, Huazhong University of Science and Technology, Wuhan 430074, China.; ^3^ China Optics Valley Laboratory, Wuhan 430074, China.; ^4^ School of Optical and Electronic Information, Huazhong University of Science and Technology, Wuhan 430074, China.

## Abstract

Lead halide perovskite nanocrystals have recently demonstrated great potential as x-ray scintillators, yet they still suffer toxicity issues, inferior light yield (LY) caused by severe self-absorption. Nontoxic bivalent europium ions (Eu^2+^) with intrinsically efficient and self-absorption-free *d–f* transition are a prospective replacement for the toxic Pb^2+^. Here, we demonstrated solution-processed organic–inorganic hybrid halide BA_10_EuI_12_ (BA denotes C_4_H_9_NH_4_^+^) single crystals for the first time. BA_10_EuI_12_ was crystallized in a monoclinic space group of *P*2_1_/*c*, with photoactive sites of [EuI_6_]^4−^ octahedra isolated by BA^+^ cations, which exhibited high photoluminescence quantum yield of 72.5% and large Stokes shift of 97 nm. These properties enable an appreciable LY value of 79.6% of LYSO (equivalent to ~27,000 photons per MeV) for BA_10_EuI_12_. Moreover, BA_10_EuI_12_ shows a short excited-state lifetime (151 ns) due to the parity-allowed *d–f* transition, which boosts the potential of BA_10_EuI_12_ for use in real-time dynamic imaging and computer tomography applications. In addition, BA_10_EuI_12_ demonstrates a decent linear scintillation response ranging from 9.21 μGy_air_ s^−1^ to 145 μGy_air_ s^−1^ and a detection limit as low as 5.83 nGy_air_ s^−1^. The x-ray imaging measurement was performed using BA_10_EuI_12_ polystyrene (PS) composite film as a scintillation screen, which exhibited clear images of objects under x-ray irradiation. The spatial resolution was determined to be 8.95 lp mm^−1^ at modulation transfer function = 0.2 for BA_10_EuI_12_/PS composite scintillation screen. We anticipate that this work will stimulate the exploration of *d–f* transition lanthanide metal halides for sensitive x-ray scintillators.

## Introduction

X-ray detectors are widely used for high-energy space physics, security inspection, oil drilling exploration, medical radiography, industrial control, and so on [1,2]. Scintillator-based indirect x-ray detection is the mainstream technology in x-ray detection applications [3]. Lead (Pb) halide perovskite nanocrystals have recently emerged as affordable alternatives to conventional ceramic scintillators [4–7]. However, their practical applications are restricted by the toxicity of the Pb^2+^, the severe self-absorption, and the complex synthesis processes.

Lanthanide (Ln: Ce–Lu) ion-based metal halides with 5*d*–4*f* transition demonstrate great promise to address the issues mentioned above because of their low toxicity, large stokes shift, high luminescence efficiency, and short decay lifetime [8–11]. Especially, the high emission efficiency resulting from the strong exciton confinement effect and negligible self-absorption of Ln^2+^ ion-based metal halides benefit high light yield (LY). The intrinsic short excited-state lifetimes of Ln^2+^ ion-based metal halides enable a fast response (~ns) under high-energy irradiation [12]. Recently, inorganic Cs_4_EuX_6_ (X = Br, I) single crystals have been reported as self-activated γ-ray scintillators with superior performance [13]. However, the crystals were prepared by the complex and expensive Bridgman method. Therefore, it is highly desired to develop Eu-based metal halide scintillators, which can be fabricated by a facile, cost-effective process.

In this work, we designed and synthesized an organic–inorganic hybrid europium (II) halide, BA_10_EuI_12_ (BA denotes C_4_H_9_NH_2_), by a facile solution method for the first time. In BA_10_EuI_12_ crystal, the photoactive sites, [EuI_6_]^4−^ octahedra, were isolated by BA^+^ cations to form a zero-dimensional (0D) crystal structure, which belonged to a monoclinic system with a space group of *Pnma* (*P*2_1_/*c*). BA_10_EuI_12_ exhibits blue emission peaked at 462 nm with narrow full width at half-maximum (FWHM) of 31 nm, a Stokes shift of 97 nm, a high photoluminescence quantum yield (PLQY) of 72.5%, and a short excited-state lifetime of 151 ns. When applied as x-ray scintillators, the BA_10_EuI_12_ crystals demonstrate a high LY value of 79.6% of LYSO (equivalent to ~27,000 photons per MeV) and possess excellent detection linear range and low detection limit under x-ray radiation.

## Results

**Fig. 1.**
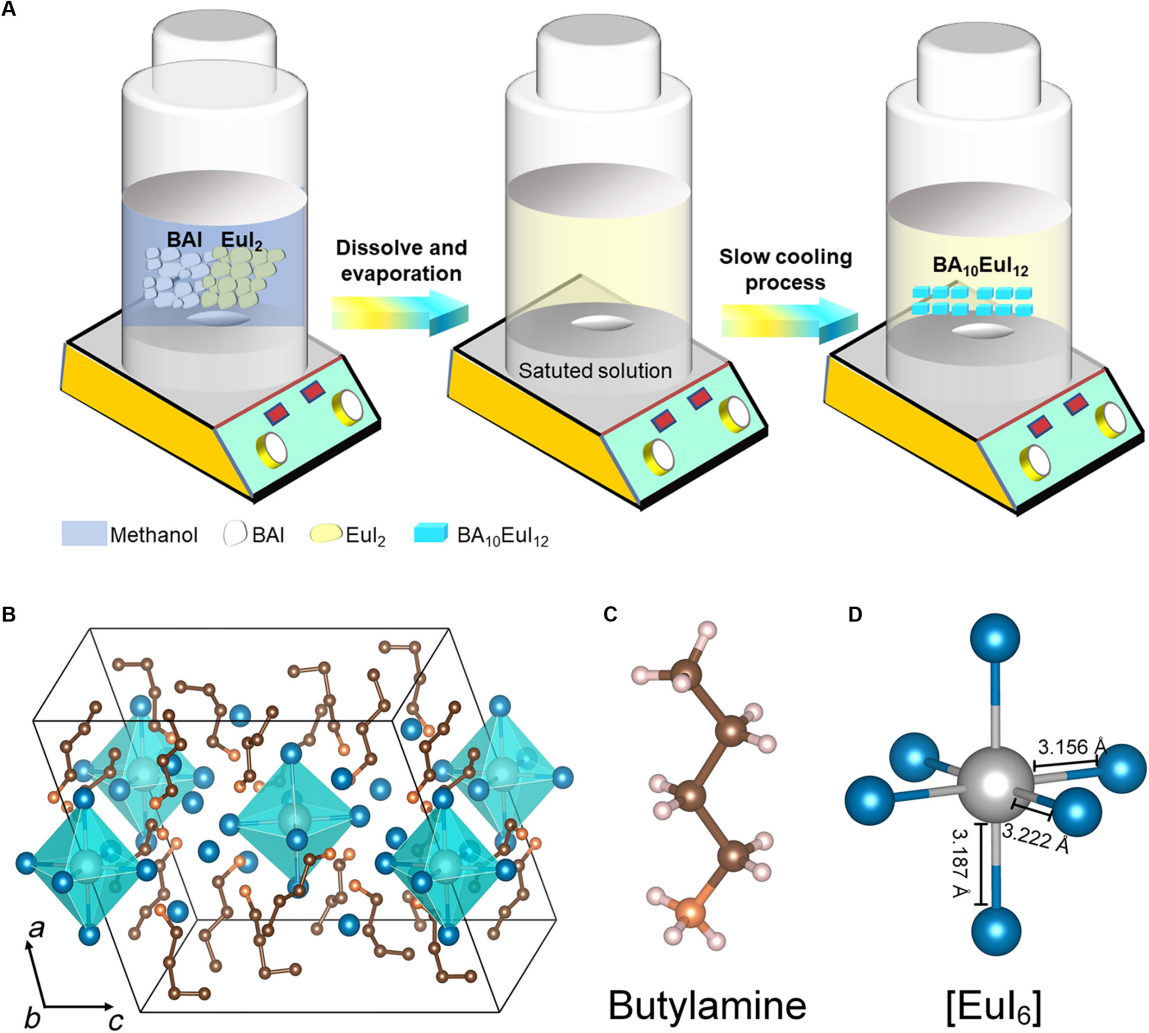
(A) Synthesis process of BA_10_EuI_12_ single crystals. (B) Crystal structure of BA_10_EuI_12_ projected along the *b* axis. Cyan octahedra correspond to [EuI_6_]^4−^, in the center of which an Eu atom (light cyan) was located and in the corners of which the I atoms (navy blue) were located. (C) Ball-and-stick model of the BA^+^ cation, in which the small orange, brown, and pink balls in the crystal structure indicate N, C, and H atoms, respectively. (D) Bond lengths of the twisted [EuI_6_]^4−^ octahedron.

BA_10_EuI_12_ crystals were synthesized by a one-step reaction from BAI and EuI_2_ through a facile solvent evaporation and temperature cooling process. Methanol was used as the solvent, in which the solubility of perovskite increases as temperature rises. The crystals were collected by first preparing a saturated solution and then slowly decreasing its temperature to enter into the oversaturation zone (Fig. 1A). The crystal structure of BA_10_EuI_12_ is determined by single-crystal x-ray diffraction (SCXRD). It crystallizes in a monoclinic space group of *P*2_1_/*c* with lattice parameters of *a* = 14.71 Å, *b* = 16.51 Å, *c* = 17.73 Å, *α* = 90°, *β* = 108.5°, *γ* = 90°, and *V* = 4,082.5 Å^3^ (Table S1). As shown in the crystal structure of BA_10_EuI_12_, the photoactive sites, [EuI_6_]^4−^ octahedra, were isolated by BA^+^ cations to form a 0D crystal structure (Fig. 1B). These isolated [EuI_6_]^4−^ octahedra are separated by layers of BA^+^ cations parallel to the (100) plane. As shown in Fig. 1C, the BA^+^ cations are larger in volume, which separate and slightly distort the [EuI_6_]^4−^ octahedra [14]. Different from EuI_2_, BA_10_EuI_12_ is 0D luminescent metal halide, which has unique crystallographic and electronic structure with fascinating optical characteristics (Fig. S1) [15,16]. Furthermore, the measured powder x-ray diffraction patterns fitted well with the simulated patterns derived from the single-crystal structure of BA_10_EuI_12_, suggesting the absence of a secondary phase and high purity of BA_10_EuI_12_ (Fig. S2). The full width at half maximum (FWHM) of the strongest diffraction peak was 0.11°, revealing good crystallinity. The x-ray photoelectron spectrum (XPS) confirmed that the europium is in the divalent state (Eu^2+^) and demonstrated the interaction between the BA^+^ cations and the central Eu^2+^. The I ion is not oxidized with the 3*d*_5/2_ and 3*d*_3/2_ peak position and doublet separation in good agreement with I^−^ (Fig. S3 and Table S2). BA_10_EuI_12_ had a low thermal decomposition temperature, which made it difficult to be prepared through the high-temperature and long-time molten salt method (Fig. S4) [17]. As a result, the facile solution method we put forward is expected to promote the development of Eu^2+^-based organic–inorganic hybrid halides.

**Fig. 2.**
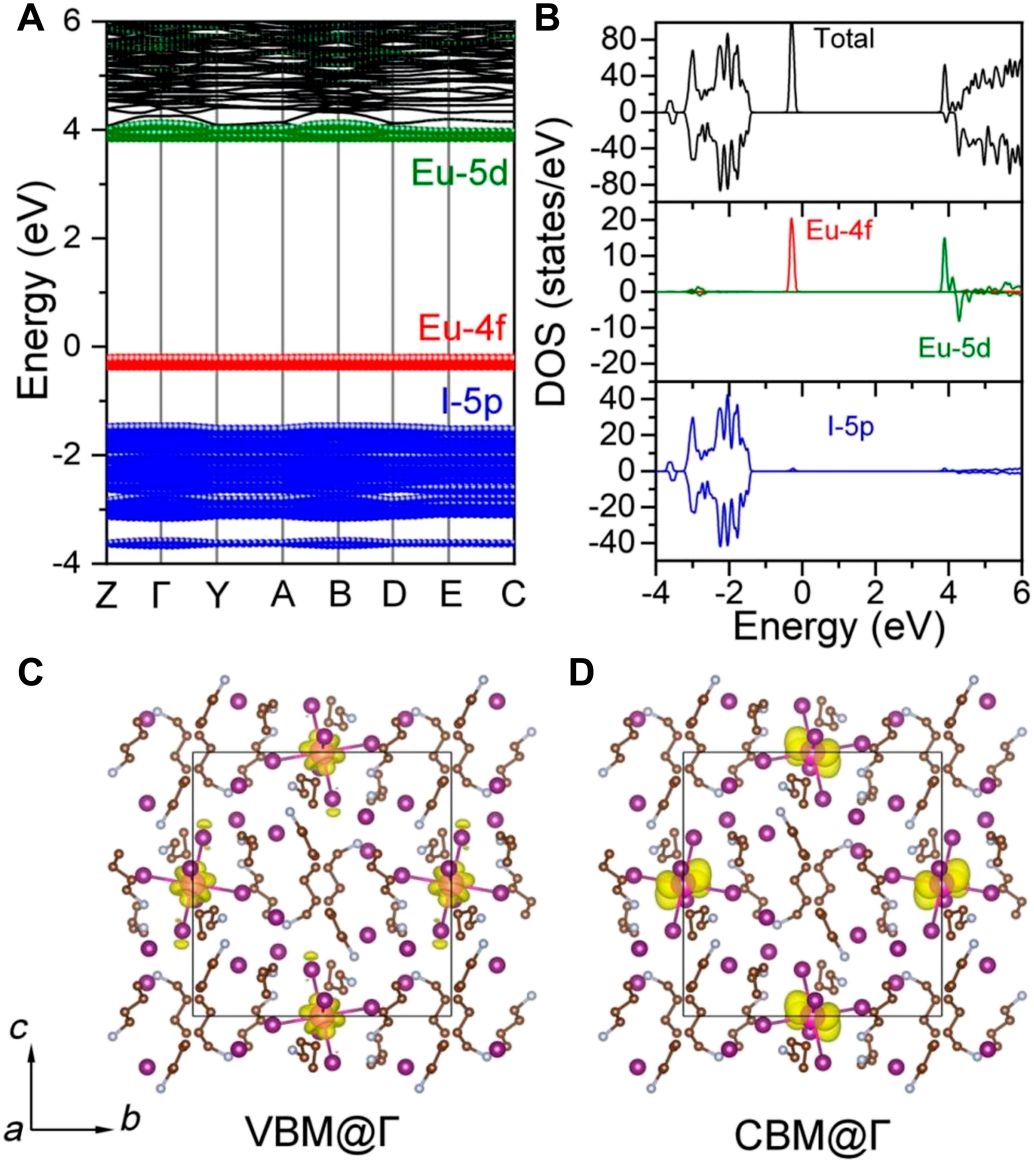
Electronic structures of BA_10_EuI_12_. (A) Calculated band structures of BA_10_EuI_12_. (B) Projected densities of states (DOS) of BA_10_EuI_12_. (C and D) Isosurface plots of the wave function |Ψ|^2^ of CBM and VBM.

First, we used density functional theory (DFT) calculations to understand the electronic structure and the luminous mechanism of BA_10_EuI_12_. The emission was caused by parity-allowed electron transitions from the 4*f*^ 6^5*d*^1^ excited states into the 4*f*^ 7^ ground states. As shown in Fig. 2A and B, the flat valence band and conduction band were attributed to the intrinsic confined 4*f* and 5*d* orbitals of Eu (II) cations and the ionic bonding nature of the Eu–I interaction. The energy of optical absorption onset resulting from excitonic absorption was slightly lower (3.06 eV), as shown in the Tauc plot (Fig. S5). The valence band maximum (VBM) is mainly attributed to the Eu-4*f* orbitals, while the conductive band minimum (CBM) is derived from Eu-5*d* orbitals (Fig. 2C and D). The organic cations do not contribute to the band edges, therefore forming a type I band alignment between [EuI_6_]^4−^ units and organic matrices, which favors efficient light emissions [18]. The electronic structure calculation reveals that the localized nature of the VBM is crucial in obtaining efficient narrow emission.

**Fig. 3.**
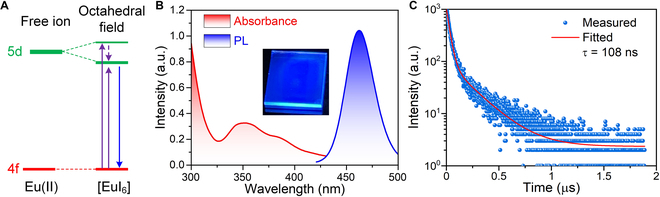
Emission mechanism and optical characterization. (A) Orbital energy diagram and the energy transition of Eu^2+^ *d*–*f* transition in the octahedral crystal field. (B) Absorbance and PL spectra of BA_10_EuI_12_. The inset presents the bright blue emission of the BA_10_EuI_12_ film under 254-nm irradiation. (C) PL lifetime of BA_10_EuI_12_. The decay curve can be well-fitted by a biexponential function.

The emission spectra of Eu^2+^ are affected by the combined effect of crystal field splitting (ε_cfs_) and centroid shift (ε_c_), which are mainly determined by the coordinating ligands (I) around Eu^2+^ and the spectroscopic polarizability α_sp_, respectively [7,12]. The weak ligand field effect in [EuI_6_] octahedra leads to little energy splitting of Eu-5*d* orbitals, which results in a large energy gap between Eu-4*f* and Eu-5*d* orbitals, and, namely, large excitation and emission energies (Fig. 3A) [19]. As shown in Fig. 3B, BA_10_EuI_12_ exhibits highly efficient pure blue photoluminescence (PL) at 462 nm with narrow FWHM (31 nm). The photoluminescence excitation (PLE) centered at 365 nm, indicating a large Stokes shift (97 nm, 0.59 eV; Fig. S6A) [20]. The low dielectric constant of the organic layers poorly screens the attraction between electrons and holes in the inorganic layers, and the inorganic layers confine the exciton’s wave function to zero dimension [21]. Thereby, the large Stokes shift results from the confined 5*d*-4*f* transitions in [EuI_6_] octahedra, consequently guaranteeing negligible peak overlap (Fig. 3B) [22]. The negligible self-absorption property together with the high theoretical exciton utilization efficiency origin from the strong exciton’s confinement effect of BA_10_EuI_12_ endows it with a high PLQY of 72.5%, which benefits high light output for scintillators (Fig. S6B). The room temperature PL decay curve was measured under 340-nm excitation by time-resolved PL measurement, and a biexponential function fitting was conducted to reveal a short PL lifetime (108 ns) of BA_10_EuI_12_ (Fig. 3C). The short lifetime boosts the potential of BA_10_EuI_12_ for use in high-energy physics and computer tomography (CT) applications. For example, modern CT requires the decay time of the scintillator to be lower than 10 μs to match the sampling rates ≥10 kHz [23].

**Fig. 4.**
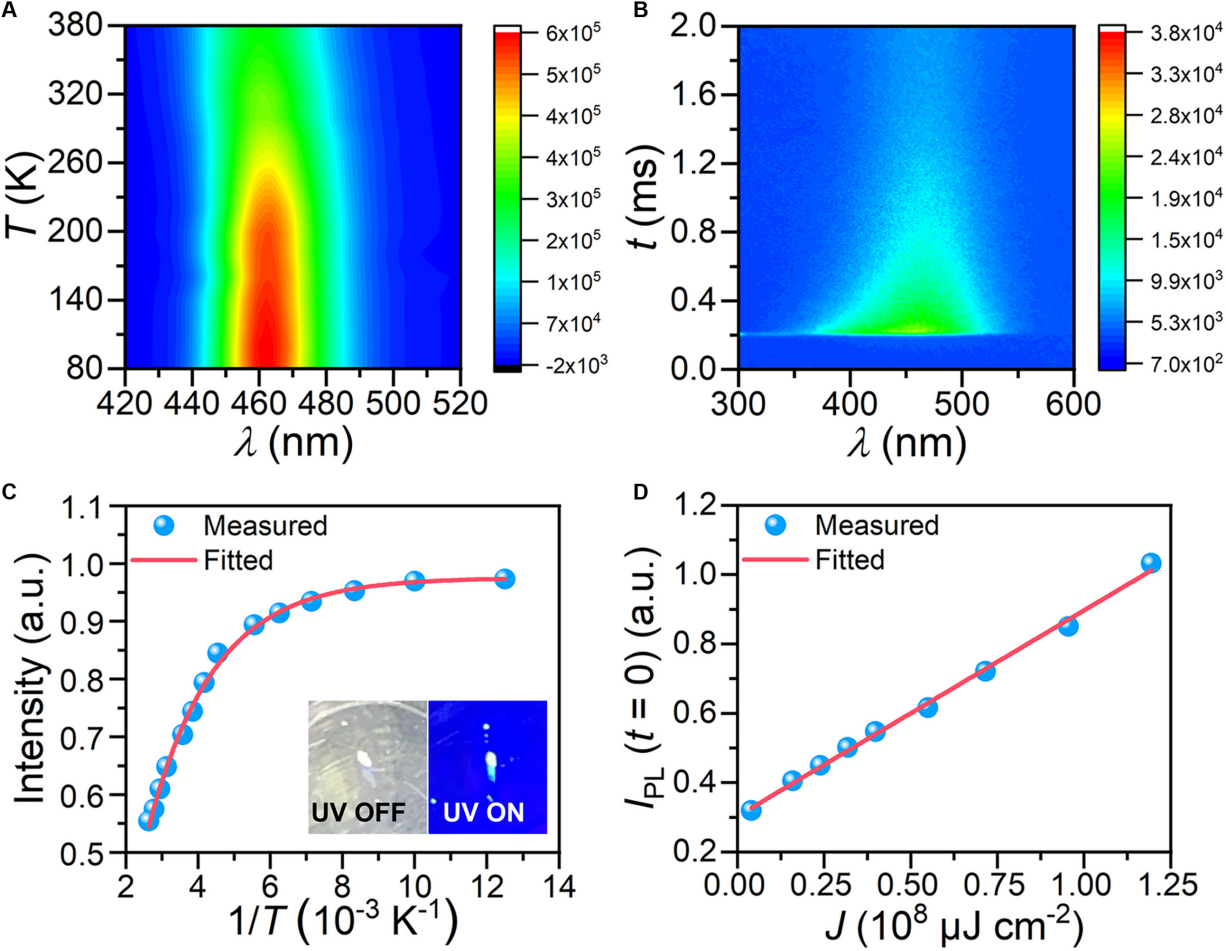
Photophysical properties of BA_10_EuI_12_. Pseudocolor map of the temperature-dependent PL spectra ranging from 80 to 380 K (A) and time-resolved fluorescence spectra (B) of BA_10_EuI_12_. (C) PL intensity derived from the temperature-dependent PL spectra as a function of temperature. The inset presents the photograph of the BA_10_EuI_12_ crystal under daylight and 254-nm irradiation. (D) PL intensity as a function of the incident intensity from 3.98 × 106 μJ cm^2^ to 1.19 × 108 μJ cm^2^.

To investigate the photophysical properties, the temperature-dependent PL spectra and time-resolved fluorescence spectra were further explored (Fig. 4A and B). The plot of PL integrated intensity [*I*(*T*)] as a function of reciprocal temperature from 80 to 380 K showed a decrease in the value of *I*(*T*) with the elevation of the temperature (Fig. 4C). The *E*_a_ value of BA_10_EuI_12_ was calculated by fitting data to Eq. 1:

$$I\left(T\right)=\frac{I_{0}}{1+\mathrm{Aexp}\left(-E_{a}/k_{B}T\right)}$$ (1)

where *E*_a_ is the thermal activation energy, *T* is the test temperature, *I*_0_ is *I*(*T*) at *T* = 0 K, and *k*_B_ is the Boltzmann constant. The *E*_a_ value is estimated to be 242 meV, indicating that thermal quenching is negligible at room temperature. As shown in Fig. 4B, BA_10_EuI_12_ demonstrated identical decay traces of the PL spectra intensity across the entire emission spectra range, indicating only one emission center for BA_10_EuI_12_. Moreover, the linear correlation between PL and incident light intensity and the constant lifetimes at different incident wavelengths indicated that the luminescence of BA_10_EuI_12_ was not from defect states, which further verified that the luminescence mechanism is the *d*–*f* transition (Fig. 4D and Figs. S7 and S8). Ln^2+^ has low stability against water and oxygen, among which Eu^2+^ has relatively high stability and low reduction potential (Eu^2+^/Eu^3+^ = 0.35 V). Fortunately, encapsulation technology can help keep the scintillators stable against water and oxygen, which has been successfully demonstrated by commercial CsI and LaBr_3_ encapsulated before their practical x-ray sensing and imaging application.

**Fig. 5.**
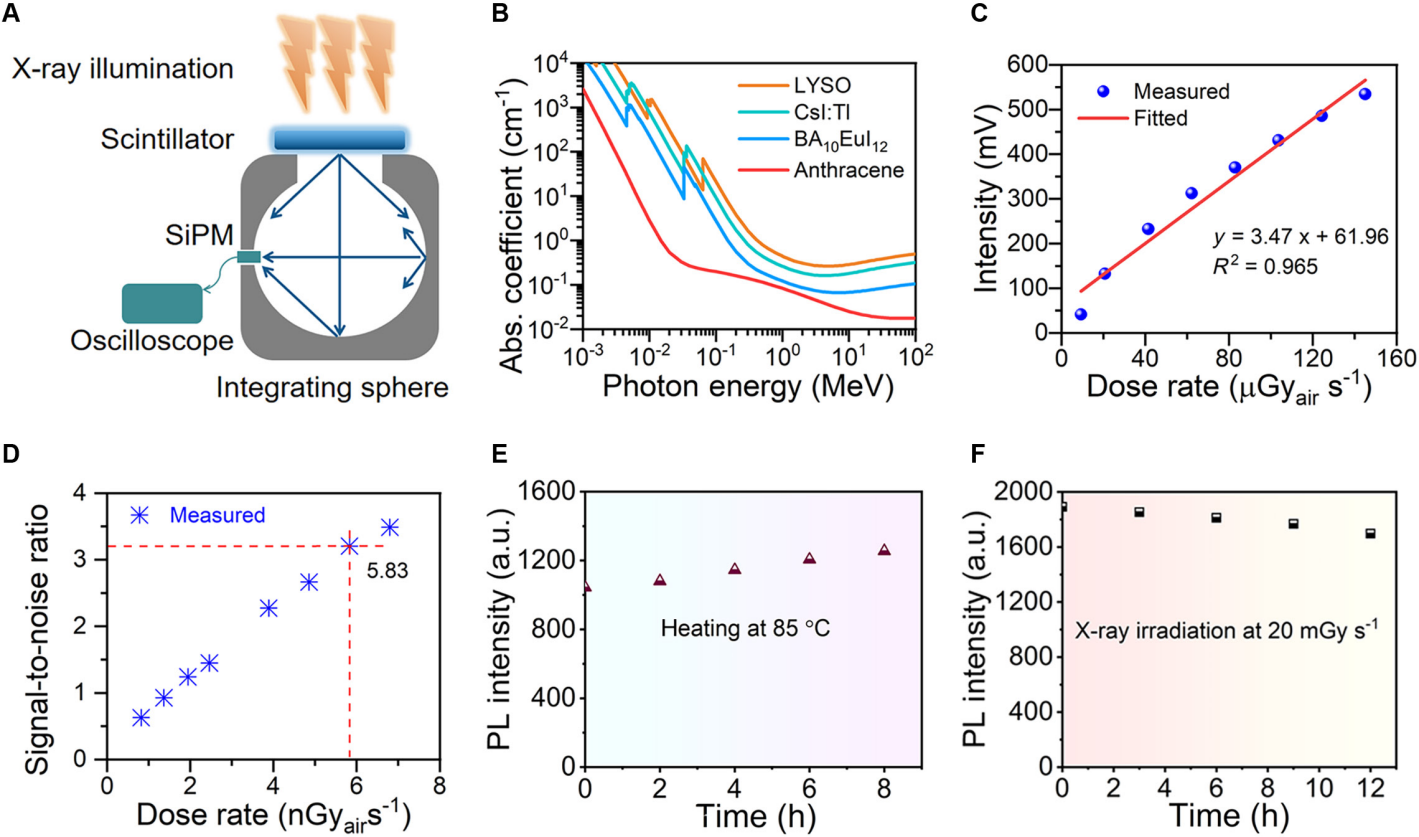
(A) Scheme of scintillator performance measurement system. (B) Absorption coefficients of BA_10_EuI_12_, CsI: Tl, LYSO, and anthracene as a function of photon energy from 1 keV (soft x-rays) to 100 MeV according to the photon cross-section database. (C) Linear response of BA_10_EuI_12_ covers an extensive range. The voltage responses of the SiPM coupled to the integrating sphere are proportional to the photon number produced by the BA_10_EuI_12_ scintillator. (D) Signal-to-noise ratio value versus the dose rate. The PL intensity of BA_10_EuI_12_ under continuous heating at 85 °C (E) and x-ray irradiation at a dose rate of 20 mGy s^−1^ with a total dosage of 864 Gy_air_ (F).

The high PLQY and short decay lifetime on the nanosecond scale make the BA_10_EuI_12_ crystal an excellent candidate for an x-ray scintillator [24,25]. The LY of BA_10_EuI_12_ was measured by using LYSO as the reference. In each test, the response of the sample was recorded by coupling it with a silicon photomultiplier (SiPM) in the integrating sphere under identical test conditions (Fig. 5A). In addition, due to the existence of heavier elements, Eu (Z = 63) and I (Z = 53), the BA_10_EuI_12_ crystals have a high x-ray attenuation ability. According to the calculated result from the software named “Auto Z_eff_,” the mean effective atomic number (Z_eff_) of BA_10_EuI_12_ is 26.65 when the incident energy is set as a 60-keV x-ray (Fig. S9) [26]. The absorption coefficient of a scintillator to x-ray is described by α∝ρZ_eff_^4^/E^3^. Accordingly, BA_10_EuI_12_ is an organic–inorganic hybrid material, which contains many light atoms such as C, H, and N. The lack of heavy elements in BA_10_EuI_12_ results in its low absorption to x-ray with the absorption coefficient inferior to the traditional scintillators such as CsI: Tl and LYSO. The x-ray absorption of BA_10_EuI_12_ is not as good as in inorganic materials such as CsPbBr_3_, but higher than the representative organic scintillators, anthracene (Fig. 5B). The BA_10_EuI_12_ crystals demonstrate LY as high as 79.6% of LYSO under irradiation with an Amptek Mini-X tube at 50-kV working voltage and 140-μA current (dose rate: 5.30 mGy_air_ s^−1^). According to a previous study, LYSO has an LY of 33,900 photons per MeV (photons MeV^−1^) [27]. Ruling out the possible impact of SiPM, the BA_10_EuI_12_ crystals have LY equivalent to ~27,000 photons MeV^−1^. There are many excellent Eu^2+^-based scintillators for gamma-ray spectroscopy with high LY. Compared with Eu^2+^-doped scintillators, BA_10_EuI_12_ has a facile fabrication method and a short fluorescence lifetime (Table S3) [12,26–31]. Lifetime is also an important factor affecting the applications of scintillators. For example, long-life scintillators have optical memory advantages to develop flat-panel-free x-ray detectors [33] and short-life scintillators are suitable for evaluating fast/real-time x-ray imaging. We define the LY value to decay time ratio as the figure of merit (FoM), also known as the initial photon rate, to evaluate the use of scintillators for fast/real-time x-ray imaging [34]. We compared BA_10_EuI_12_ with several representative scintillators (Table S4 and Fig. S10) [30–35]. Although the FoM of BA_10_EuI_12_ scintillator is 179 photons MeV^−1^ ns^−1^ and lower than LaBr_3_: Ce (LaBr_3_: 5% Ce 4,000 photons MeV^−1^ ns^−1^; LaBr_3_: 0.2% Ce 1,867 photons MeV^−1^ ns^−1^), it is higher than most scintillators [23,34,36–38]. As shown in Fig. 5C, the dose-dependent response of BA_10_EuI_12_ was measured, demonstrating a linear response to the x-ray dose ranging from 9.21 μGy_air_ s^−1^ to 145 μGy_air_ s^−1^. As shown in Fig. 5D, the detection limit of BA_10_EuI_12_ is as low as 5.83 nGy_air_ s^−1^, which is much lower than that of LYSO (3.5 μGy_air_ s^−1^) [27]. The low detection limit of BA_10_EuI_12_ is approximately 1,000 times lower than the dosage for a standard medical diagnostic (5.5 μGy a time), which could reduce the radiation dosage used for medical examination and thus alleviate the carcinogenic risks during diagnosis [39]. Moreover, BA_10_EuI_12_ has good thermal and irradiation stability with negligible loss of emission intensity of BA_10_EuI_12_ PL strength under continuous heating at 85 °C in the glove box and continuous x-ray irradiation (Fig. 5E and F and Fig. S11). Compared with the common Eu^2+^ emitter scintillators, BA_10_EuI_12_ is prepared through the facile solution method (Table S4).

**Fig. 6.**
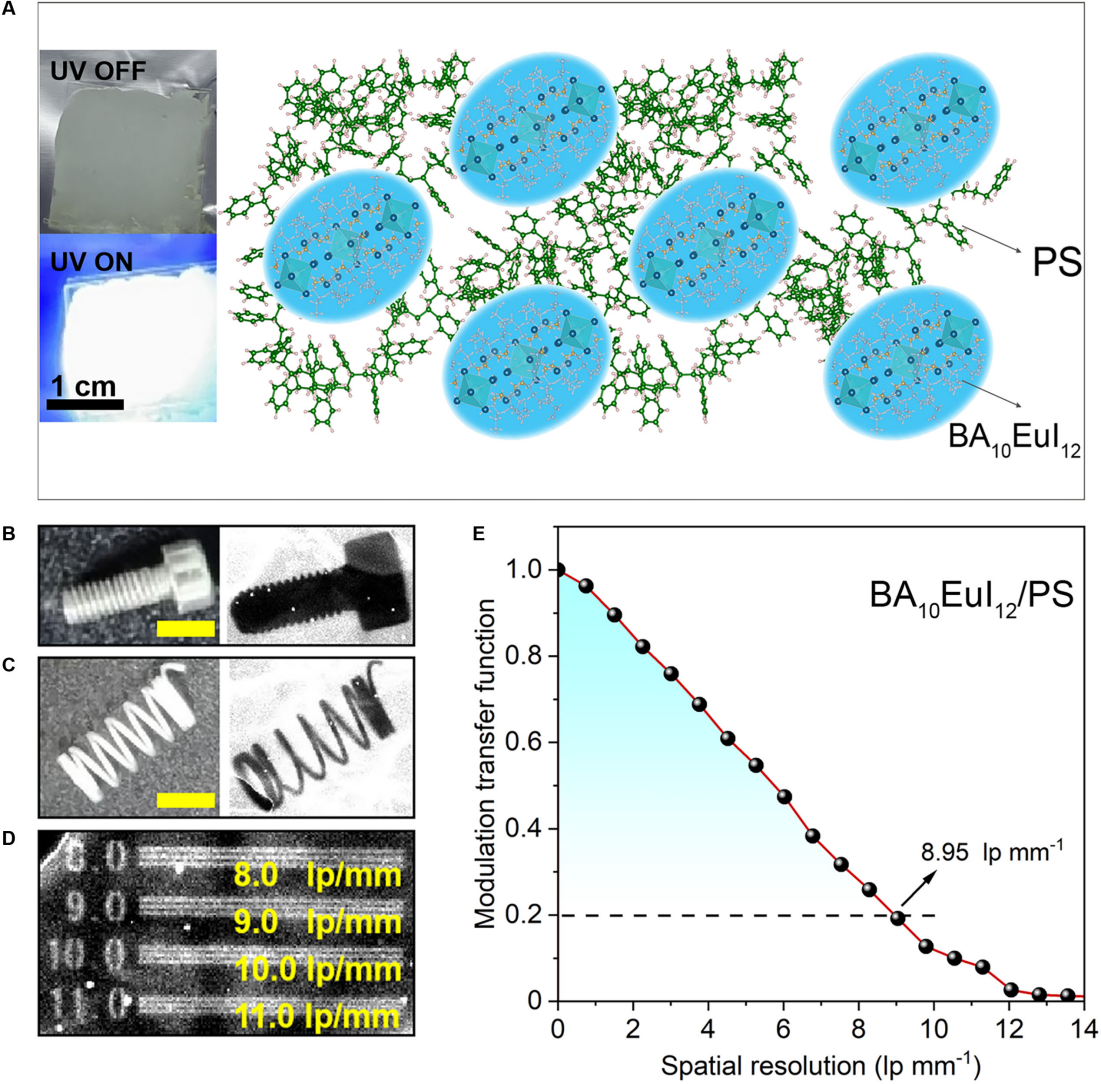
(A) Schematic illustration of mixing BA_10_EuI_12_ and PS to fabricate scintillator thick films. The green atoms denote benzene rings of PS, and the cyan octahedra correspond to [EuI_6_]^4−^. The inset presents the photograph of the BA_10_EuI_12_/PS film under daylight and 254-nm irradiation. (B and C) Photographs and x-ray images of a stainless nail and a spring. Scale bars, 5 mm. (D) X-ray images of a partial region (from 8 to 11 lp mm^−1^) of the standard x-ray test pattern (x-ray tube voltage: 60 kV, 200 mA; dose rate: 2.86 mGy_air_ s^−1^). (E) MTFs of x-ray image obtained from the BA_10_EuI_12_/PS scintillator film. The spatial resolution (when the MTF value equals 0.2) is 8.95 lp mm^−1^.

Inspired by the promising scintillation property of BA_10_EuI_12_, we investigated the possibility of demonstrating practical x-ray imaging through a homemade optical system (Fig. S12). To make a free-standing scintillator screen, we mixed the BA_10_EuI_12_ scintillators with polystyrene (PS) to obtain a free-standing film with BA_10_EuI_12_ crystals embedded inside the PS matrix. The PS is the host matrix to fabricate a uniform thick film without causing emission quenching and the encapsulation to protect BA_10_EuI_12_ from atmospheric water and oxygen (Fig. 6A). The photographs and x-ray images of a stainless nail and spring are shown in Fig. 6B and C, respectively. To evaluate the spatial resolution, we took images of the pattern plate for the standard x-ray resolution test (Fig. 6D), showing that the observation limit was between ≈8 and 10 lp mm^−1^. To further confirm those values of BA_10_EuI_12_/PS, we determined the modulation transfer function (MTF; expressed in units of line pairs per millimeter) through the slanted-edge method for a quantitative resolution value. The spatial resolution of the BA_10_EuI_12_/PS film was determined to be 8.95 lp mm^−1^ at MTF = 0.2, consistent with its x-ray resolution pattern test result (Fig. 6E). The high resolution of BA_10_EuI_12_/PS film was comparable with the reported good-performing scintillators (Table S5) [36,40–48]. We believe that these results will stimulate further work on Eu-based scintillators for x-ray imaging.

## Discussion

In summary, we have successfully developed the BA_10_EuI_12_ scintillator through a facile solution processing method. BA_10_EuI_12_ showed blue emission with negligible self-absorption, a high PLQY of 72.5%, and a short excited-state lifetime (108 ns). The BA_10_EuI_12_ scintillators had the characteristics of nontoxicity, easy fabrication, decent LY value of 79.6% of LYSO (equivalent to ~27,000 photons MeV^−1^), excellent linear response to x-ray dose rate, and a low detection limit. As a result, high-resolution x-ray imaging of 8.95 lp mm^−1^ was realized. It is anticipated that this work could inspire the *d*–*f* transition Ln ion-based metal halides for promising x-ray scintillators and neutrons and γ-ray detection in the future.

## Materials and Methods

### Chemicals

N-butyl amine (C_4_H_9_NH_2_·HI, BAI, 99.5%) was purchased from Xi'an Polymer Light Technology Co. Ltd. Europium (II) iodide (EuI_2_, 99.9 wt%) was purchased from Tianjin Novcare Biotechnology Co. Ltd. Dried methanol (CH_3_OH, 99.0%) was purchased from J and K Scientific Reagent Co. Ltd. All reagents and solvents were used without further purification.

### BA_10_EuI_12_ single-crystal synthesis

The BA_10_EuI_12_ single crystals were synthesized by a one-step reaction between BAI and EuI_2_ through a solvent evaporation process followed by a slow cooling process. BAI (0.5 mol l^−1^) and EuI_2_ (0.5 mol l^−1^) are solved in anhydrous methanol in glass bottles. Then, the vial containing the stoichiometric amount of BAI and EuI_2_ was stirred and heated at 50 °C under an inert N_2_ atmosphere until forming a saturation precursor solution. The clear solution was then cooled to room temperature (25 °C) at a rate of 5 °C h^−1^. BA_10_EuI_12_ was crystallized gradually with the decrease of solubility during the cooling process, and the BA_10_EuI_12_ crystals were washed with antisolvent anhydrous methylbenzene and followed by vacuumed drying.

### BA_10_EuI_12_/PS composite film synthesis

First, 2 g of PS (average *M*_w_ ~ 280,000) was dissolved in 10 ml of anhydrous toluene by stirring at room temperature to form a transparent solution. Second, BA_10_EuI_12_ single-crystal powders were added to the solution and stirred thoroughly. Finally, the uniform composites were coated on glass and vacuum-dried.

### Inert protection

The BA_10_EuI_12_ single crystal was encapsulated by using vacuum grease during the SCXRD test. For luminescent properties and x-ray imaging test, the BA_10_EuI_12_ and BA_10_EuI_12_/PS films were encapsulate by 2 pieces of thin glass (0.2 mm in thickness) followed by the application of ultraviolet (UV)-curable adhesive.

### Characterizations

SCXRD data of BA_10_EuI_12_ were collected using an XtaLAB PRO MM007HF diffractometer with Cu Kα radiation. The crystal was mounted in the sample holder surrounded with vacuum silicone and held at 110 K for data collection. The subsequent crystal structure determination and refinement were carried out using CRYSTALS. Structural characterization was conducted by using powder X-ray diffraction (PXRD) recorded on a Philips diffractometer (X pert pro-MRD) operating at 40 kV and 40 mA with Cu Kα radiation. The XPS spectra measurements were conducted using the Thermo Scientific K-Alpha+ from ThermoFisher. The thermogravimetric analysis and differential scanning calorimetry (TGA-DSC) was performed with a PerkinElmer Instruments, Diamond TG/DSC6300, to determine the thermal stability of BA_10_EuI_12_. The UV–visible absorption spectra were collected with a UV–vis–near-IR spectrophotometer (Shimadzu Instruments, SolidSpec-3700). The PLE, PL, and time-resolved PL spectra were measured by the Edinburgh FLS920 system. PLQY was obtained through Hamamatsu Quantaurus-QY, with the excitation wavelength at 365 nm. The PL decay spectra were recorded using the Edinburgh FLS920 system with a 340-nm laser beam as the light source at room temperature. The temperature-dependent PL spectra were also measured using the Edinburgh FLS920 system. The selected single crystal was put into the sample room (Linkam attachment, temperature range: 77 to 600 K).

### Computational methods

DFT calculations were performed using the Vienna Ab initio Simulation Package (VASP) 6.1 code [49] with the projection-augmented wave (PAW) method. The plane-wave cutoff energy was set to 400 eV. The structural optimization was performed using the Heyd-Scuseria-Ernzerhof (HSE) [50,51] hybrid functionals with a mixing parameter of 25%. Only Γ point was employed for sampling the Brillouin zones, and crystal structures were fully relaxed until the total force on each atom was <0.03 eV/Å.

### Calculation of Z_eff_

The Z_eff_ value of BA_10_EuI_12_ was determined using Auto-Zeff software developed by Taylor et al. [26]. After imputing the formula as parameter and setting a certain incident energy, the Z_eff_ value can be obtained.

### X-ray scintillation measurement

An Amptek Mini-X tube (Au target, 10 W, Newton Scientific M237), an integrating sphere, a SiPM (JSP-TN3050-SMT), and an oscilloscope (Keysight) were used. The typical scintillator LYSO was selected as the reference for the scintillator property calculation. The samples were placed in the integrating sphere to measure the emission intensity and obtain the LY accurately. In this way, we can eliminate test errors due to more severe light scattering of LYSO than the BA_10_EuI_12_ crystal, which leads the scintillation light to come out from LYSO without coupling into SiPM because of the optical waveguide effect. Moreover, we encapsulated BA_10_EuI_12_ with a polymer film, made it have the same shape as LYSO, and placed the samples in the same position to obtain the LY value accurately.

According to the proportion between the LY and the test signal, the LY of BA_10_EuI_12_ is obtained by taking the known LY of LYSO and different irradiation areas into account. In detail, the LY of BA_10_EuI_12_ can be obtained from Eq. 2:

$$\begin{array}{c} \frac{{\mathrm{LY}}_{\mathrm{BA}10\mathrm{EuI}12}}{{\mathrm{LY}}_{\mathrm{LYSO}}}=\frac{R_{\mathrm{BA}10\mathrm{EuI}12}}{R_{\mathrm{LYSO}}}\times \\ \frac{\int I_{\mathrm{LYSO}}\left(\lambda \right)S\left(\lambda \right)d\lambda /\int I_{\mathrm{LYSO}}\left(\lambda \right)d\lambda }{\int I_{\mathrm{BA}10\mathrm{EuI}12}\left(\lambda \right)S\left(\lambda \right)d\lambda /\int I_{\mathrm{BA}10\mathrm{EuI}12}\left(\lambda \right)d\lambda }\times \frac{S_{\mathrm{LYSO}}}{S_{\mathrm{BA}10\mathrm{EuI}12}} \end{array}$$ (2)

where *R* is the voltage response, *I* (*λ*) is the radioluminescence (RL) intensity, and *S* (*λ*) is the detection efficiency of SiPM. *S*_LYSO_ and *S*_BA10EuI12_ are the area exposed to x-ray of LYSO and BA_10_EuI_12_, respectively. As for the measurement of the linear range, the samples generated light proportionally under x-ray irradiation with a series of intensities. The dose rate is calibrated by an ion chamber dosimeter (MagicMax from IBA DOSIMETRY).

### X-ray imaging

We assembled a homemade imaging system in a black lead box. The x-ray source used 435 in the system was M237 (50 kV, Newton Scientific) with Au target. The BA_10_EuI_12_/PS film was placed on a reflector (CCM1-G01, Thorlabs), and the scintillation light was deflected to a 437 CMOS camera (C13440, Hamamatsu) with a pixel size of 6.5 × 6.5 μm^2^.

## Data Availability

Data used to support the findings of this study are included within the article and supplementary information files.

## References

[1] Gandini M, Villa I, Beretta M, Gotti C, Imran M, Carulli F, Fantuzzi E, Sassi M, Zaffalon M, Brofferio C, et al. Efficient, fast and reabsorption-free perovskite nanocrystal-based sensitized plastic scintillators. Nat Nanotechnol. 2020;15(6):462–468. 32424340 10.1038/s41565-020-0683-8

[2] Liu SP, Mares JA, Feng XQ, Vedda A, Fasoli M, Shi Y, Kou HM, Beitlerova A, Wu LX, D'Ambrosio C, et al. Towards bright and fast Lu_3_Al_5_O_12_:Ce,Mg optical ceramics scintillators. Adv Opt Mater. 2016;4(5):731–739.

[3] Chen Q, Wu J, Ou X, Huang B, Almutlaq J, Zhumekenov AA, Guan X, Han S, Liang L, Yi Z, et al. All-inorganic perovskite nanocrystal scintillators. Nature. 2018;561(7721):88–93. 30150772 10.1038/s41586-018-0451-1

[4] Liu C, Li Z, Hajagos TJ, Kishpaugh D, Chen DY, Pei Q. Transparent ultra-high-loading quantum dot/polymer nanocomposite monolith for gamma scintillation. ACS Nano. 2017;11(6):6422–6430. 28551988 10.1021/acsnano.7b02923

[5] He Y, Matei L, Jung HJ, McCall KM, Chen M, Stoumpos CC, Liu Z, Peters JA, Chung DY, Wessels BW, et al. High spectral resolution of gamma-rays at room temperature by perovskite CsPbBr_3_ single crystals. Nat Commun. 2018;9(9):1609. 29686385 10.1038/s41467-018-04073-3 PMC5913317

[6] Wang Y, Yin X, Liu W, Xie J, Chen J, Silver MA, Sheng D, Chen L, Diwu J, Liu N, et al. Emergence of uranium as a distinct metal center for building intrinsic X-ray scintillators. Angew Chem Int Ed Engl. 2018;57(26):7883–7887. 29600818 10.1002/anie.201802865

[7] Zhang Y, Sun R, Qi X, Fu K, Chen Q, Ding Y, Xu L, Liu L, Han Y, Malko AV, et al. Metal halide perovskite nanosheet for X-ray high-resolution scintillation imaging screens. ACS Nano. 2019;13(2):2520–2525. 30721023 10.1021/acsnano.8b09484

[8] Luo J, Yang L, Tan Z, Xie W, Sun Q, Li J, Du P, Xiao Q, Wang L, Zhao X, et al. Efficient blue light emitting diodes based on europium halide perovskites. Adv Mater. 2021;33(38):Article e2101903. 34342910 10.1002/adma.202101903

[9] Dorenbos P, van Loef EVD, Vink AP, van der Kolk E, van Eijk CWE, Kramer KW, Gudel HU, Higgins WM, Shah KS. Level location and spectroscopy of Ce^3+^, Pr^3+^, Er^3+^, and Eu^2+^ in LaBr_3_. J Lumin. 2006;117(2):147–155.

[10] López-Lugo VH, Rivera-Medina MJ, Alonso-Huitrón JC. Quantitative assessing of crystal field, nephelauxetic, and Stokes shift effects on the blue luminescence of Eu^2+^ ions incorporated in ZnS films. Mater Res Express. 2021;8:Article 036406.

[11] Li J, Wang L, Zhao Z, Sun B, Zhan G, Liu H, Bian Z, Liu Z. Highly efficient and air-stable Eu(II)-containing azacryptates ready for organic light-emitting diodes. Nat Commun. 2020;11:5218. 33060573 10.1038/s41467-020-19027-x PMC7562750

[12] Bizarri G, Bourret-Courchesne ED, Yan Z, Derenzo SE. Scintillation and optical properties of BaBrI:Eu^2+^ and CsBa_2_I_5_:Eu^2+^. IEEE Trans Nucl Sci. 2011;58(6):3403–3410.

[13] Wu Y, Han D, Chakoumakos BC, Shi H, Chen S, Du M-H, Greeley I, Loyd M, Rutstrom DJ, Stand L, et al. Zero-dimensional Cs_4_EuX_6_ (X = Br, I) all-inorganic perovskite single crystals for gamma-ray spectroscopy. J Mater Chem C. 2018;6(25):6647–6655.

[14] Li L, Sun Z, Wang P, Hu W, Wang S, Ji C, Hong M, Luo J. Tailored engineering of an unusual (C_4_H_9_NH_3_)_2_ (CH_3_NH_3_)_2_Pb_3_Br_10_ two-dimensional multilayered perovskite ferroelectric for a high-performance photodetector. Angew Chem Int Ed Engl. 2017;56(40):12150–12154. 28719061 10.1002/anie.201705836

[15] Li M, Xia Z. Recent progress of zero-dimensional luminescent metal halides. Chem Soc Rev. 2021;50(4):2626–2662. 33399145 10.1039/d0cs00779j

[16] Han K, Jin J, Su B, Xia Z. Molecular dimensionality and photoluminescence of hybrid metal halides. Trends Chem. 2022;4:1034–1044.

[17] Mitzi DB, Liang KN. Preparation and properties of (C_4_H_9_NH_3_)_2_EuI_4_: A luminescent organic−inorganic perovskite with a divalent rare-earth metal halide framework. Chem Mater. 1997;9(12):2990–2995.

[18] Fu PF, Sun YL, Xia ZG, Xiao ZW. Photoluminescence behavior of zero-dimensional manganese halide tetrahedra embedded in conjugated organic matrices. J Phys Chem Lett. 2021;12(31):7394–7399. 34328337 10.1021/acs.jpclett.1c02154

[19] Yang L, Luo J, Gao L, Song B, Tang J. Inorganic lanthanide compounds with f–d transition: From materials to electroluminescence devices. J Phys Chem Lett. 2022;13:4365–4373. 35544383 10.1021/acs.jpclett.2c00927

[20] Franck J, Dymond EG. Elementary processes of photochemical reactions. Trans Faraday Soc. 1926;21(21):536–542.

[21] Smith MD, Karunadasa HI. White-light emission from layered halide perovskites. Acc Chem Res. 2018;51(3):619–627. 29461806 10.1021/acs.accounts.7b00433

[22] Wang S, Song Z, Kong Y, Xia Z, Liu Q. Crystal field splitting of 4f^n−1^ 5d-levels of Ce^3+^ and Eu^2+^ in nitride compounds. J Lumin. 2018;194:461–466.

[23] Lecoq P. Development of new scintillators for medical applications. Nucl Instrum Meth Phys Res Sect A. 2016;809:130–139.

[24] Perego J, Villa I, Pedrini A, Padovani EC, Crapanzano R, Vedda A, Dujardin C, Bezuidenhout CX, Bracco S, Sozzani PE, et al. Composite fast scintillators based on high-Z fluorescent metal–organic framework nanocrystals. Nat Photon. 2021;15:393–400.

[25] Guo Q, Zhao X, Song B, Luo J, Tang J. Light emission of self-trapped excitons in inorganic metal halides for optoelectronic applications. Adv Mater. 2022;Article e2201008. 35322473 10.1002/adma.202201008

[26] Taylor ML, Smith RL, Dossing F, Franich RD. Robust calculation of effective atomic numbers: The Auto-Z(eff) software. Med Phys. 2012;39:1769–1778. 22482600 10.1118/1.3689810

[27] Zhao X, Niu G, Zhu J, Yang B, Yuan JH, Li S, Gao W, Hu Q, Yin L, Xue KH, et al. All-inorganic copper halide as a stable and self-absorption-free X-ray scintillator. J Phys Chem Lett. 2020;11(5):1873–1880. 32040318 10.1021/acs.jpclett.0c00161

[28] Boatner LA, Ramey JO, Kolopus JA, Hawrami R, Higgins WM, van Loef E, Glodo J, Shah KS, Rowe E, Bhattacharya P, et al. Bridgman growth of large SrI_2_:Eu^2+^ single crystals: A high-performance scintillator for radiation detection applications. J Cryst Growth. 2013;379:63–68.

[29] Alekhin MS, de Haas JTM, Kraemer KW, Dorenbos P. Scintillation properties of and self absorption in SrI_2_:Eu^2+^. IEEE Trans Nucl Sci. 2011;58:2519–2527.

[30] Wang S, Rutstrom DJ, Stand L, Koschan M, Melcher CL, Wu Y. Optical and scintillation properties of Hf^ 4+^ codoped SrI_2_:Eu^2+^ single crystals. IEEE Trans Nucl Sci. 2020;67(6):876–879.

[31] Bourret-Courchesne ED, Bizarri G, Borade R, Yan Z, Hanrahan SM, Gundiah G, Chaudhry A, Canning A, Derenzo SE. Eu^2+^-doped Ba_2_CsI_5_, a new high-performance scintillator. Nucl Instrum Meth Phys Res Sect A. 2009;612(1):138–142.

[32] Wu Y, Li Q, Jones S, Dun C, Hu S, Zhuravleva M, Lindsey AC, Stand L, Loyd M, Koschan M, et al. Defect engineering by codoping in KCaI3:Eu^2+^ single-crystalline scintillators. Phys Rev Appl. 2017;8:Article 034001.

[33] Ou X, Qin X, Huang B, Zan J, Wu Q, Hong Z, Xie L, Bian H, Yi Z, Chen X, et al. High-resolution X-ray luminescence extension imaging. Nature. 2021;590:410–415. 33597760 10.1038/s41586-021-03251-6

[34] Zhang H, Liu X, Dong J, Yu H, Zhou C, Zhang B, Xu Y, Jie W. Centimeter-sized inorganic lead halide perovskite CsPbBr_3_ crystals grown by an improved solution method. Cryst Growth Des. 2017;17(12):6426–6431.

[35] Weber MJ. Scintillation: Mechanisms and new crystals. Nucl Instrum Methods Phys Res Sect A. 2004;527(1–2):9–14.

[36] Zhao X, Jin T, Gao WR, Niu GD, Zhu JS, Song BX, Luo JJ, Pan WC, Wu HD, Zhang MY, et al. Embedding Cs_3_Cu_2_I_5_ scintillators into anodic aluminum oxide matrix for high-resolution X-ray imaging. Adv Opt Mater. 2021;9(24):Article 2101194.

[37] Zhang M, Zhu J, Yang B, Niu G, Wu H, Zhao X, Yin L, Jin T, Liang X, Tang J. Oriented-structured CsCu_2_I_3_ film by close-space sublimation and nanoscale seed screening for high-resolution X-ray imaging. Nano Lett. 2021;21(3):1392–1399. 33480701 10.1021/acs.nanolett.0c04197

[38] Bizarri G, Dorenbos P. Charge carrier and exciton dynamics in LaBr_3_: Ce^3+^ scintillators: Experiment and model. Phys Rev B. 2007;75(18):Article 184302.

[39] Maddalena F, Tjahjana L, Xie AZ, Arramel SW, Zeng H, Wang P, Coquet W, Drozdowski C, Dujardin C, Dang MD, et al. Inorganic, organic, and perovskite halides with nanotechnology for high–light yield X- and γ-ray scintillators. Crystals. 2019;9(2):88–117.

[40] Zhao J, Zhao L, Deng Y, Xiao X, Ni Z, Xu S, Huang J. Perovskite-filled membranes for flexible and large-area direct-conversion X-ray detector arrays. Nat Photon. 2020;14:612–617.

[41] Han K, Sakhatskyi K, Jin J, Zhang Q, Kovalenko MV, Xia Z. Seed-crystal-induced cold sintering toward metal halide transparent ceramic scintillators. Adv Mater. 2022;34(17):Article e2110420. 35231955 10.1002/adma.202110420

[42] Han K, Jin JC, Su BB, Qiao JW, Xia ZG. Promoting single channel photon emission in copper(I) halide clusters for X-ray detection. Adv Opt Mater. 2022;10(20):2200865.

[43] Ma W, Jiang T, Yang Z, Zhang H, Su Y, Chen Z, Chen X, Ma Y, Zhu W, Yu X, et al. Highly resolved and robust dynamic X-ray imaging using perovskite glass-ceramic scintillator with reduced light scattering. Adv Sci. 2021;8:2003728. 10.1002/advs.202003728 PMC8336613 34075729

[44] Wei H, Huang J. Halide lead perovskites for ionizing radiation detection. Nat Commun. 2019;10:1066. 30842411 10.1038/s41467-019-08981-w PMC6403296

[45] Nagarkar VV, Gupta TK, Miller SR, Klugerman Y, Squillante MR, Entine G. Structured CsI(Tl) scintillators for X-ray imaging applications. IEEE Trans Nucl Sci. 1998;45(3):492–496.

[46] Wang S, Lei YT, Chen HY, Peng GQ, Wang Q, Wang HX, Duan JL, Jin ZW. Vertically oriented porous PET as template to integrated metal halide for high-performance large-area and ultra-flexible X-Ray detector. Small. 2022;18(52):2205095. 10.1002/smll.202205095 36373681

[47] Zhang F, Zhou YC, Chen ZP, Wang M, Ma ZZ, Chen X, Jia MC, Wu D, Xiao JW, Li XJ, et al. Thermally activated delayed fluorescence zirconium-based perovskites for large-area and ultraflexible X-ray scintillator screens. Adv Mater. 2022;34(43):2204801. 10.1002/adma.202204801 36047911

[48] Wang JX, Gutierrez-Arzaluz L, Wang XJ, He TY, Zhang YH, Eddaoudi M, Bakr OM, Mohammed OF. Heavy-atom engineering of thermally activated delayed fluorophores for high-performance X-ray imaging scintillators. Nat Photon. 2022;16:869–875.

[49] Kresse F, Furthmüller J. Efficient iterative schemes for ab initio total-energy calculations using a plane-wave basis set. Phys Rev B. 1996;54(16):11169–11186. 10.1103/physrevb.54.11169 9984901

[50] Heyd J, Scuseria GE, Ernzerhof M. Hybrid functionals based on a screened Coulomb potential. J Chem Phys. 2003;118(18):8207–8215.

[51] Heyd J, Scuseria GE, Ernzerhof M. Erratum: “Hybrid functionals based on a screened Coulomb potential” [J. Chem. Phys. 118, 8207 (2003)]. J Chem Phys. 2006;124(21):Article 219906.

