# Supplementary material for: Solution-Processed Hybrid Europium (II) Iodide Scintillator for Sensitive X-Ray Detection
Source: Research (Wash D C). 2023 May 5;6:0125. doi: 10.34133/research.0125 (PMC10202385; doi:10.34133/research.0125)
Supplement: Supplementary Materials — Tables S1 to S5 Figs. S1 to S12 Data file S1 [file research.0125.f1.docx]

Supporting Information

Solution-Processed Hybrid Europium (II) Iodide Scintillator for Sensitive X-Ray Detection

Xue Zhao†^1,2^, Pengfei Fu†^2^, Pan Li^2^, Hainan Du^2^, Jinsong Zhu^2^, Ciyu Ge^2^, Longbo Yang^2^, Boxiang Song^2^, Haodi Wu^2^*, Tong Jin^2^, Qingxun Guo^2^, Liang Wang^2^, Jinghui Li^2^, Zewen Xiao^2^*, Jiajun Luo^2^*, Guangda Niu^2,3^, Jiang Tang^2,3,4^

^1^ School of Microelectronics, Xidian University, Xi’an 710071, China.

^2^ Wuhan National Laboratory for Optoelectronics, Huazhong University of Science and Technology, Wuhan 430074, China.

^3^China Optics Valley Laboratory, Wuhan 430074, China.

^4^School of Optical and Electronic Information, Huazhong University of Science and Technology, Wuhan 430074, China.

Correspondence should be addressed to Jiajun Luo; [luojiajun@hust.edu.cn](mailto:luojiajun@hust.edu.cn); Zewen Xiao; [zwxiao@hust.edu.cn](mailto:zwxiao@hust.edu.cn%20) and Haodi Wu; [whd18501@163.com](mailto:whd18501@163.com)

**Table S1.** Crystallographic data for BA_10_EuI_12_

| Identification code | BA_10_EuI_12_ |
| --- | --- |
| Empirical formula | C_40_H_120_N_10_EuI_12_ |
| Formula weight/ g mol^-1^ | 2416.21 |
| Temperature/K | 100.00(10) |
| Crystal system | monoclinic |
| Space group | *P*2_1_/*c* |
| *a*/Å | 14.7070(6) |
| *b*/Å | 16.5078(4) |
| *c*/Å | 17.7315(5) |
| *α*/° | 90 |
| *β*/° | 108.497(4) |
| *γ*/° | 90 |
| Volume/Å^3^ | 4082.5(2) |
| Z | 2 |
| ρ_calc_/g cm^-3^ | 1.966 |
| μ/mm^-1^ | 41.338 |
| *F*(000) | 2258.0 |
| Crystal size/mm^3^ | 0.5 × 0.4 × 0.3 |
| Radiation | Cu Kα (λ = 1.54184) |
| 2Θ range for data collection/° | 6.338 to 103.046 |
| Index ranges | -14 ≤ *h* ≤ 14  -16 ≤ *k* ≤ 16  -17 ≤ *l* ≤12 |
| Reflections collected | 13765 |
| Independent reflections | 4433 [R_int_ = 0.0853, R_sigma_ = 0.0917] |
| Data/restraints/parameters | 4433/126/296 |
| Goodness-of-fit on F^2^ | 1.048 |
| Final R indexes [I>=2σ (I)] | R_1_ = 0.0680, wR_2_ = 0.1852 |
| Final R indexes [all data] | R_1_ = 0.0917, wR_2_ = 0.2012 |
| Largest diff. peak/hole /e Å^-3^ | 1.53/-1.28 |


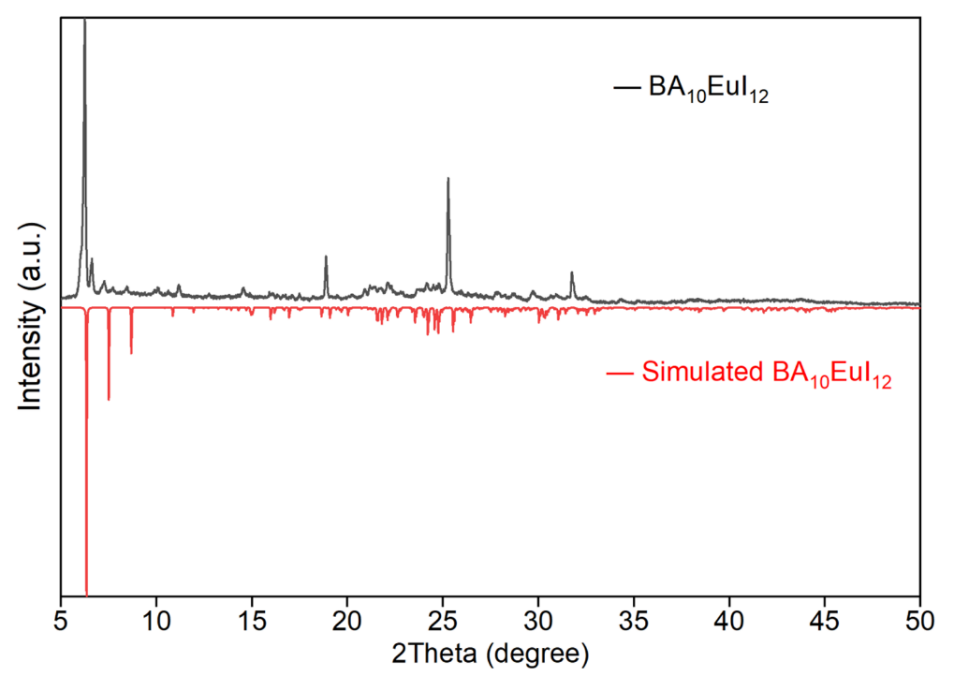


**Figure S1.** The experiment measured (black) and simulated (red) XRD patterns of BA_10_EuI_12_.


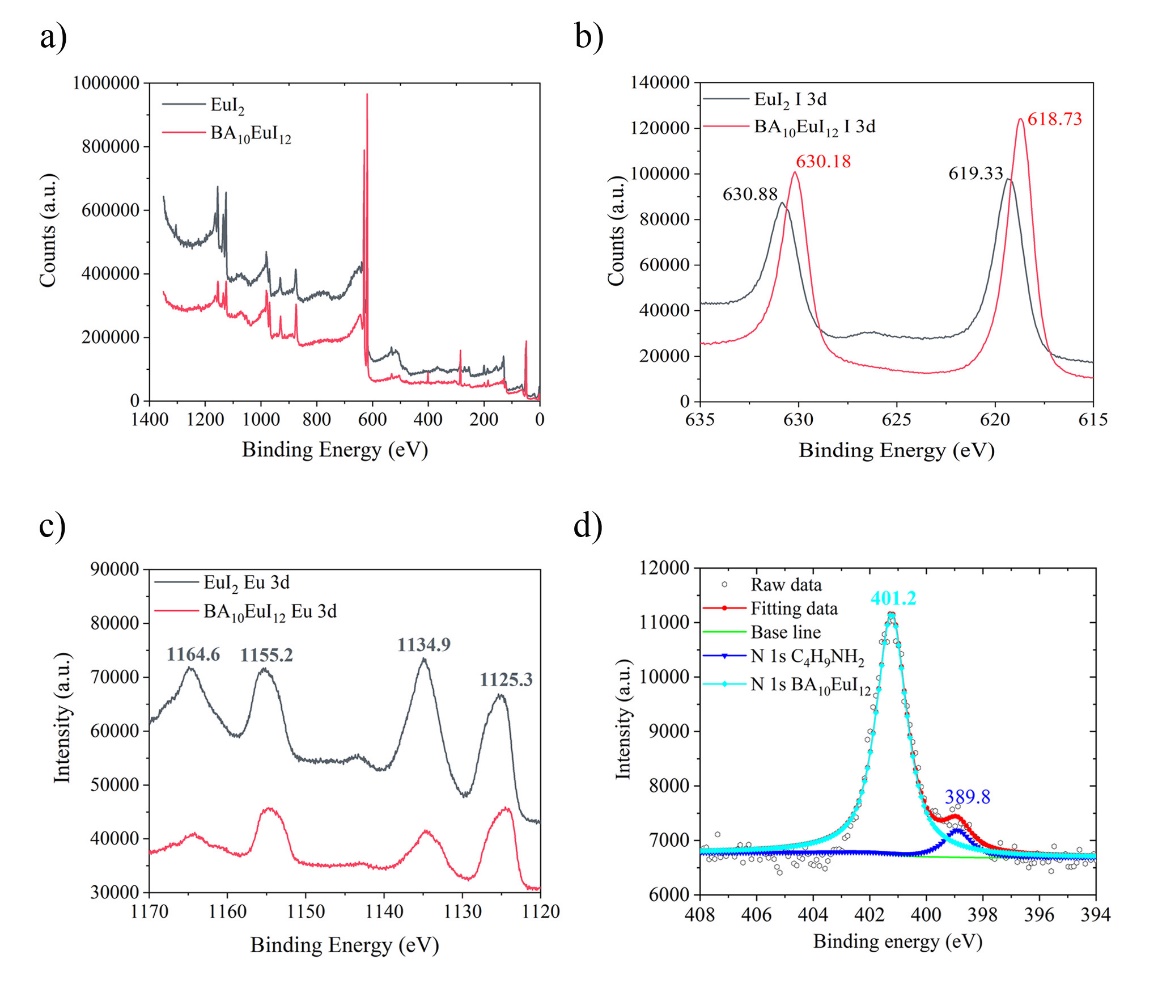


**Figure S2.** The X-ray photoelectron spectrum survey spectra (a) and high-resolution spectra of I 3*d* (b), Eu 3*d* (c), and N 1*s* (d) of BA_10_EuI_12_.

**Table S2.** The elements ratio of BA_10_EuI_12_ crystal calculated from XPS data.

| Element orbit | I 3*d* | N 1*s* | C 1*s* | Eu 3*d* |
| --- | --- | --- | --- | --- |
| Element ratio (at %) | 20.67 | 17.85 | 58.86 | 2.62 |


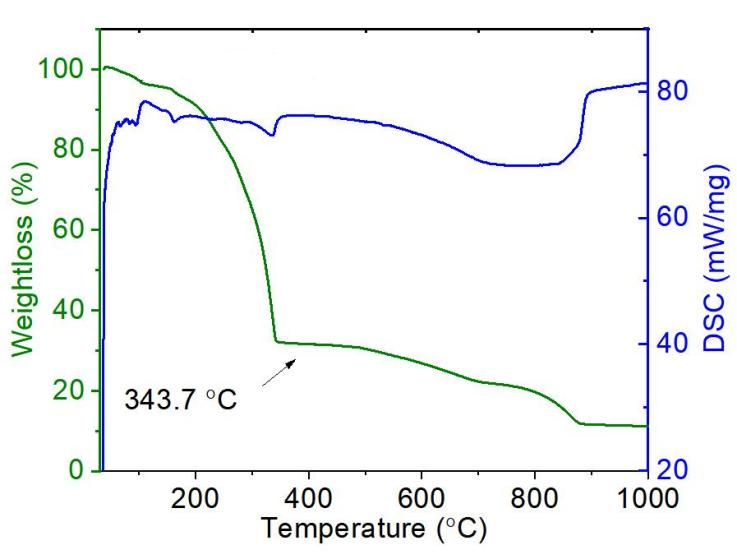


**Figure S3.** The thermogravimetric analysis (TGA) and differential scanning calorimetry (DSC) results of BA_10_EuI_12_. The experimental weight loss determined by TGA is about 70 % from room temperature to 343.7 ^o^C, which is due to the theoretical decomposition of organic BAI and in line with its weight ratio in BA_10_EuI_12_, verifying the obtained crystal structure is reliable.


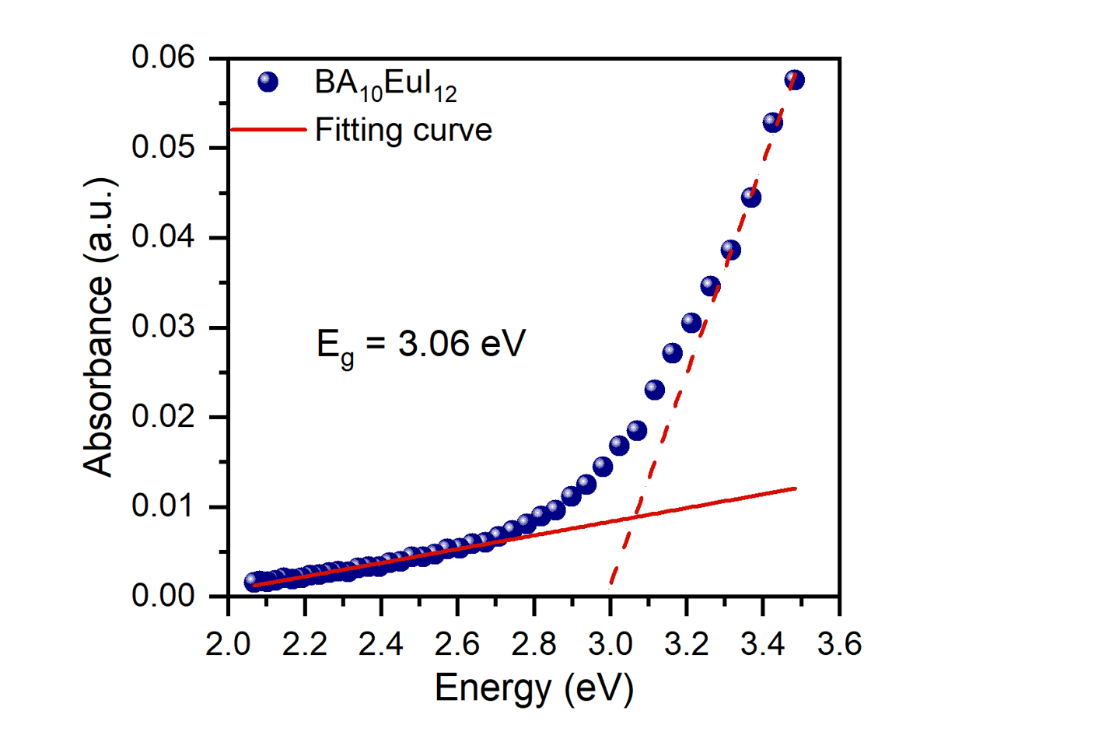


**Figure** **S4.** Tauc plot showing the bandgap of 3.06 eV for BA_10_EuI_12_ derived from UV-vis absorption curve.


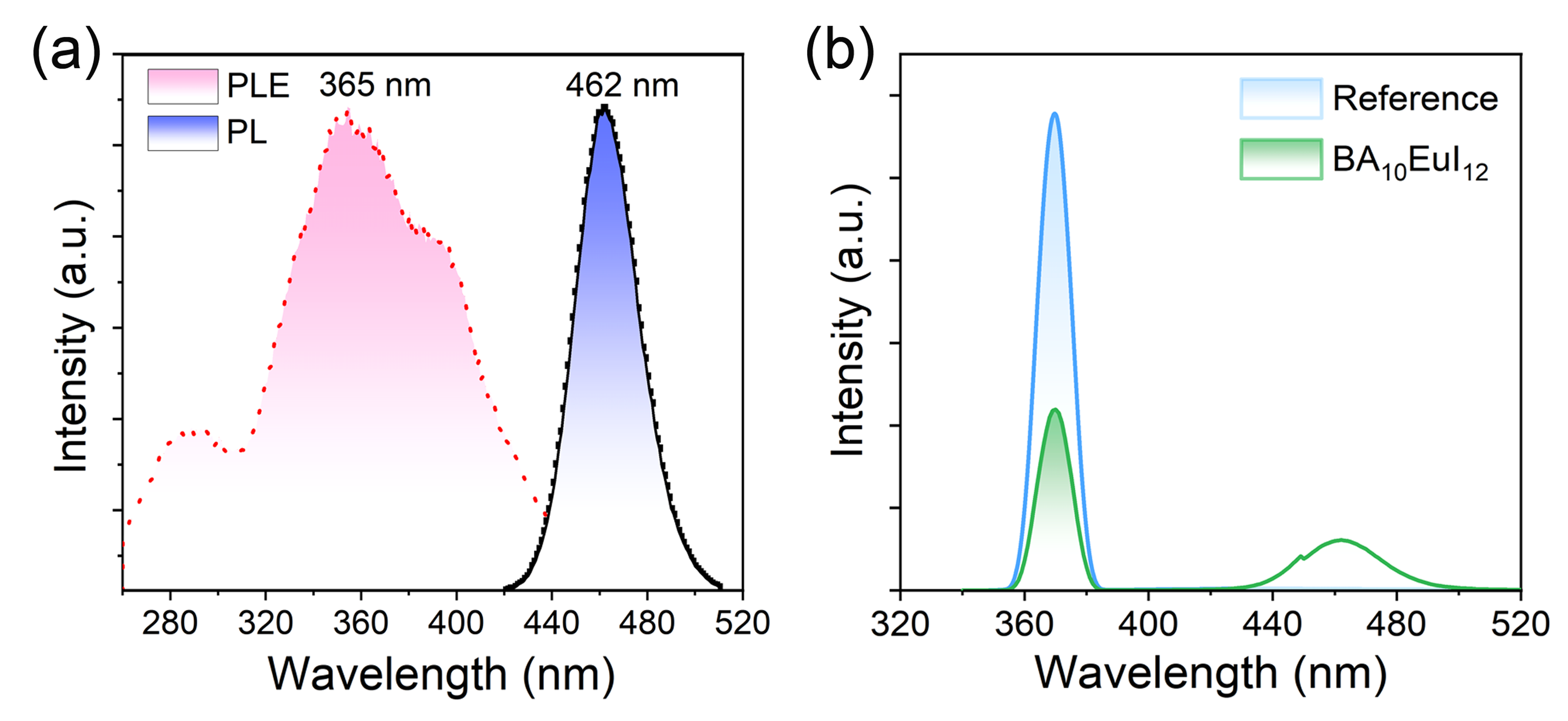


**Figure S5.** a) The photoluminescence (pink), and photoluminescence excitation (blue) spectra of BA_10_EuI_12_. b)The photoluminescence quantum yield curves of BA_10_EuI_12_.


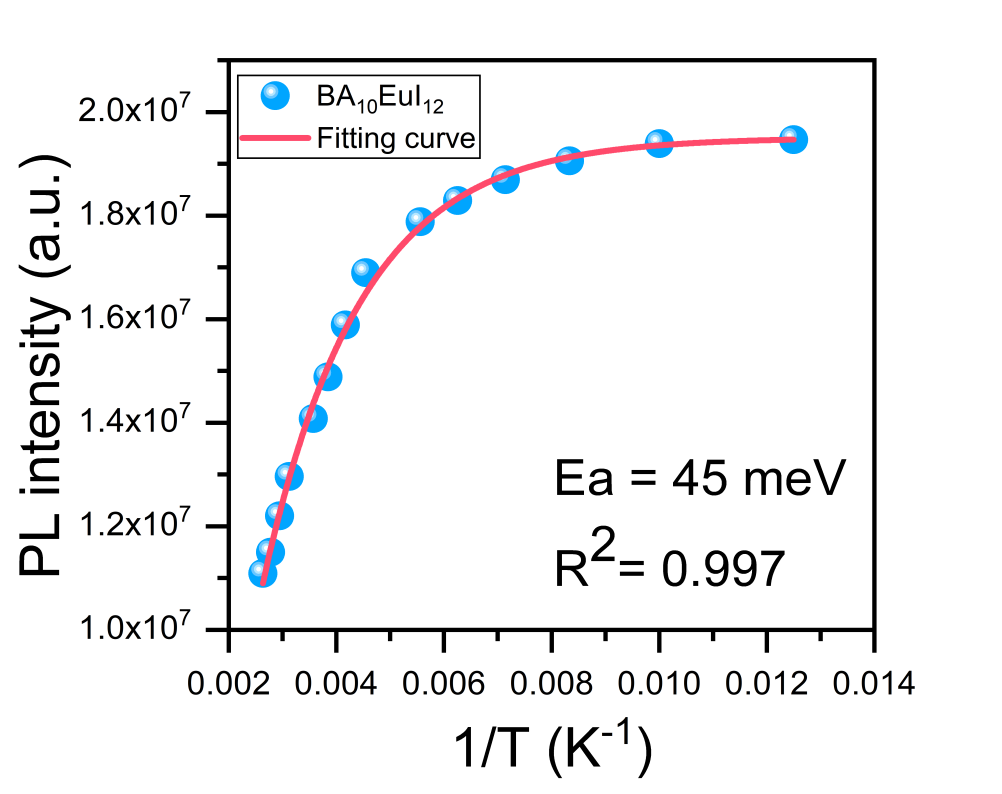


**Figure S6.** The PL integrated intensity as a function of reciprocal temperature from 80 K to 380 K.


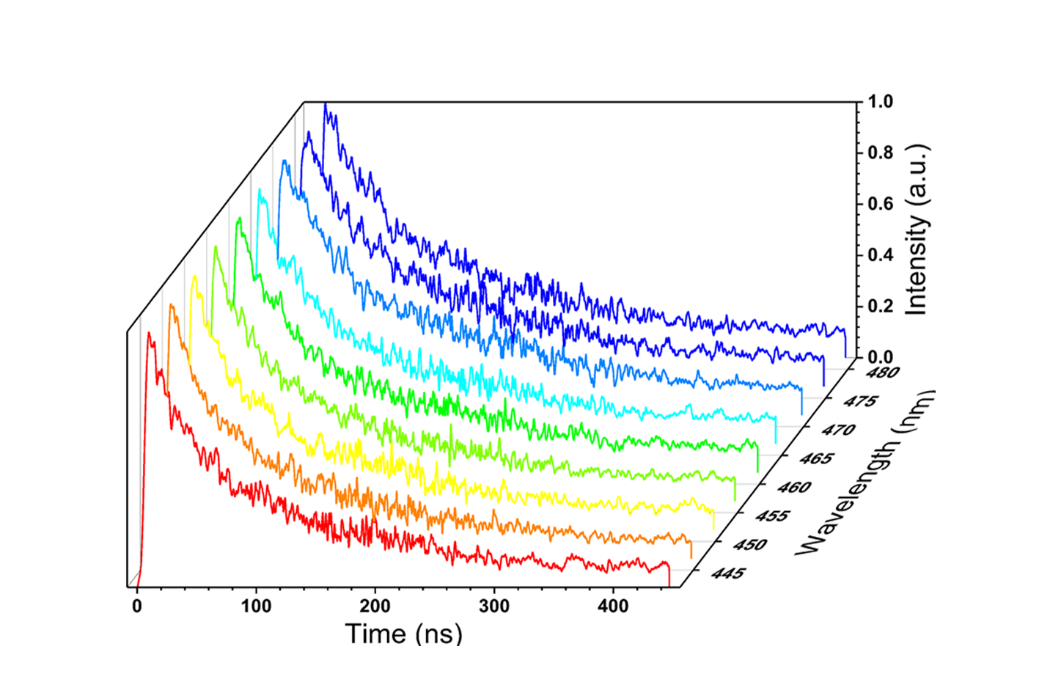


**Figure S7.** The PL intensity as a function of the incident intensity from 3.98×10^6^ μJ cm^2^ to 1.19×10^8^ μJ cm^2^.


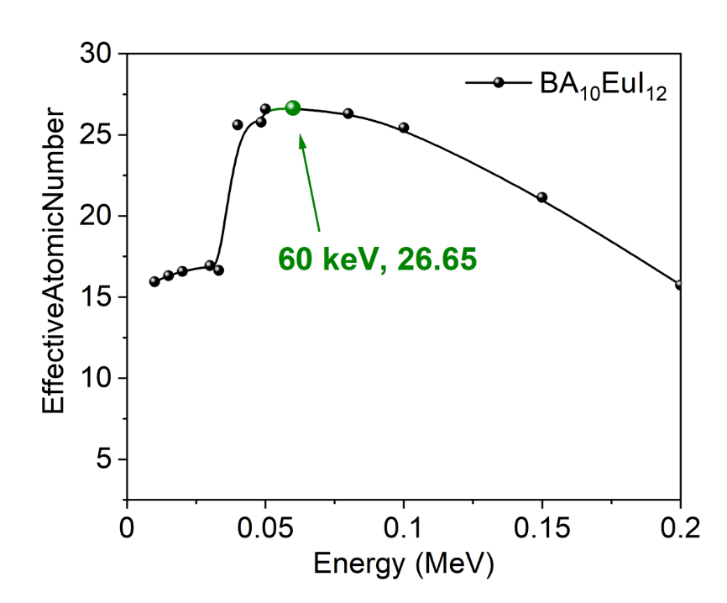


**Figure S8.** The effective atomic number of BA_10_EuI_12_.

**Table S3.** The comparison of the FoM between BA_10_EuI_12_ and several well-acknowledged scintillators.

| Scintillators | Light yield  (photons MeV^-1^) | Fluorescence lifetime (ns) | Light yield to lifetime ratio  (photons MeV^-1^ ns^-1^) | Reference |
| --- | --- | --- | --- | --- |
| LaBr_3_:5% Ce^3+^ | 64 000 | 16 | 4000 | Ref. 30 |
| LaBr_3_:0.2% Ce^3+^ | 56000 | 30 | 1867 | Ref. 30 |
| SrI_2_:Eu^2+^ | 115000 | 1200 | 96 | Ref. 30 |
| CsI: Tl | 54000 | 1000 | 54 | Ref. 30 |
| GOS | 50000 | 600000 | 7 | Ref. 30 |
| CdWO_4_ | 20000 | 5000 | 4 | Ref. 20 |
| BGO | 10000 | 300 | 33 | Ref. 20 |
| Cs_3_Cu_2_I_5_ | 29000 | 936.39 | 4 | Ref. 28 |
| BA_10_EuI_12_ | 27000 | 151 | 179 | This work |





**Figure S9**. The bar chart of the FoM between BA_10_EuI_12_ and several well-acknowledged scintillators.


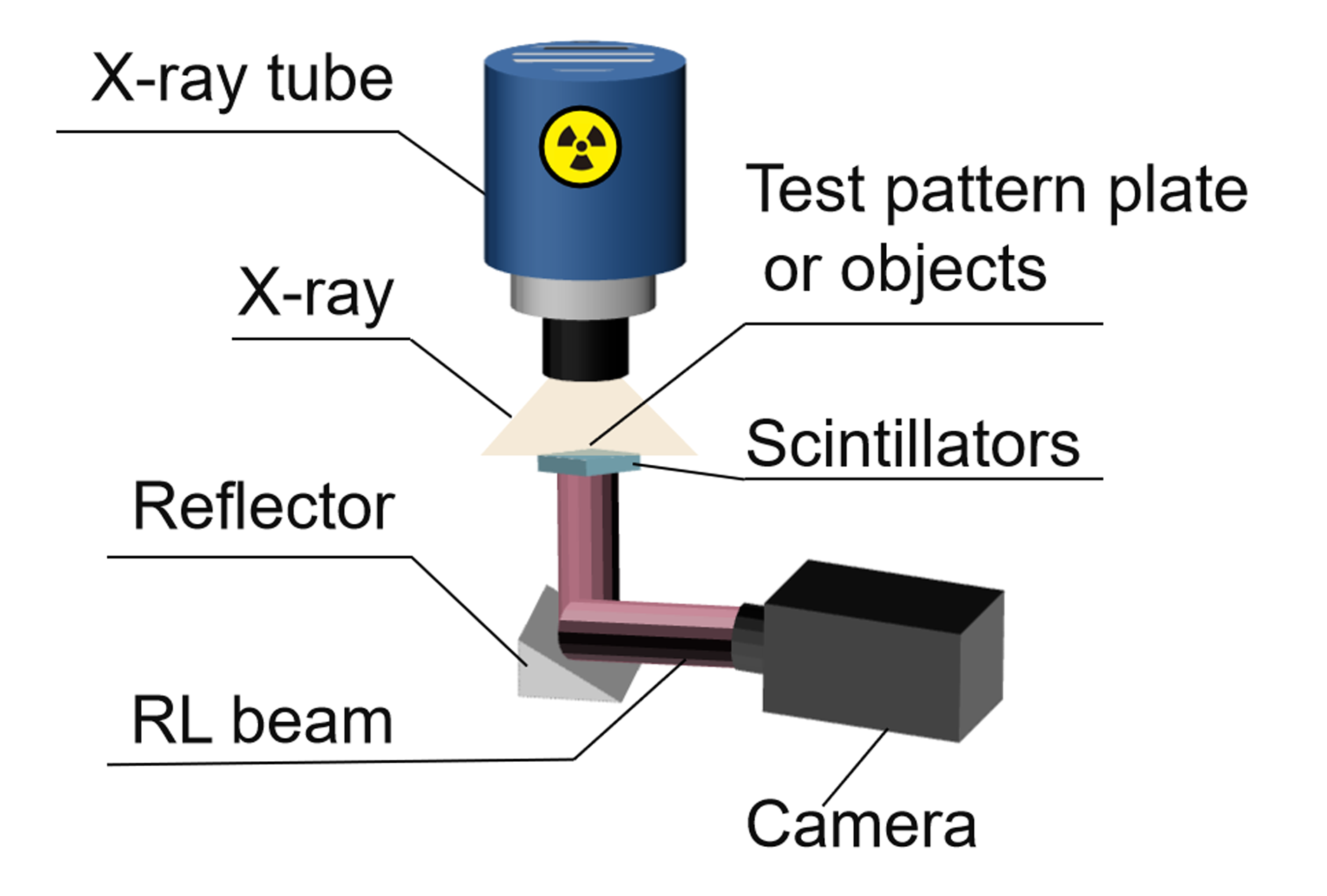


**Figure S10**. Scheme of X-ray imaging performance measurement system.

**Data file S1.** CCDC 2207129 contains the supplementary crystallographic data for this paper. These data can be obtained free of charge from The Cambridge Crystallographic Data Centre via www.ccdc.cam.ac.uk/data_request/cif.
